# Draft genome sequence of *Listeria aquatica* strain SG_BD1, isolated from a cow dung sample in Bangladesh

**DOI:** 10.1128/mra.00729-24

**Published:** 2024-09-26

**Authors:** Supantha Rivu, Elspeth Smith, Graham Stafford, Sangita Ahmed

**Affiliations:** 1 Department of Microbiology, University of Dhaka, Dhaka, Bangladesh; 2 School of Clinical Dentistry, University of Sheffield, Sheffield, United Kingdom; Queens College Department of Biology, Queens, New York, USA

**Keywords:** *Listeria aquatica*, genome analysis

## Abstract

Here, we present the genome of *Listeria aquatica* strain SG_BD1, isolated from cow dung in Dhaka, Bangladesh, and assembled after Oxford Nanopore sequencing. The genome is 2,690,148 bp with 2,855 predicted coding DNA sequences, G + C content of 39.6%, and displays a putative virulence gene *clpP* and 9 CRISPRs.

## ANNOUNCEMENT

*Listeria aquatica* is a non-pathogenic species of the genus *Listeria,* which is usually found in aquatic sources. With the ability to ferment D-tagatose and the inability to ferment maltose, this species is unique in the *Listeria* genus (1). Moreover, multiple virulence and resistance genes with close homology to the pathogenic *Listeria monocytogenes* have been detected in this species (2). In Bangladesh, the presence of *L. monocytogenes* is evident in food and environmental samples, though there is no information regarding other species (3, 4). Here, we share the draft genome sequence of *L. aquatica* strain SG_BD1, which was isolated from cow dung in Dhaka, Bangladesh in 2023.

The cow dung sample was collected from a cattle farm in Dhaka (23°43′55.2972″ N, 90°28′32.0916″ E) and transferred immediately to the Department of Microbiology, University of Dhaka. The sample was enriched in Listeria Enrichment Broth (Oxoid, England), followed by streaking on Listeria Selective Agar (Oxoid, England) and incubation at 37°C for 48 h. A black colony from Listeria selective agar was confirmed as a potential *Listeria* spp. microscopically. Using the boiled DNA method, genomic DNA was extracted from an overnight culture of the isolate grown in nutrient broth and incubated at 37°C (5) and was subjected to Oxford Nanopore MinION genome sequencing using the Oxford Nanopore Sequencing Technologies, UK-Native Barcoding Kit 24 V14 and R10.4.1 SpotON flow cells. No shearing or size selection of DNA was performed before the sequencing.

Sequencing generated 2.81 M reads with *N*_50_ of 3.59 kb. Reads below the minimum quality score value of 8 were classified as failed reads. Sequencing was analyzed using the MinKNOW GUI 5.9.17 followed by assembly using Raven Galaxy version 1.8.0 and annotation utilizing NCBI Prokaryotic Genome Annotation Pipeline version 6.7 (PGAP) (6–8). CRISPRCasFinder web server version 4.2.20 (9) was used to detect CRISPR arrays, while virulence and antibiotic resistance genes were detected using the Virulence Factor Database and Comprehensive Antibiotic Resistance Database in ABRicate 1.0.1 on Galaxy, respectively (10–12). All these tools were applied using default parameters.

**Fig 1:**
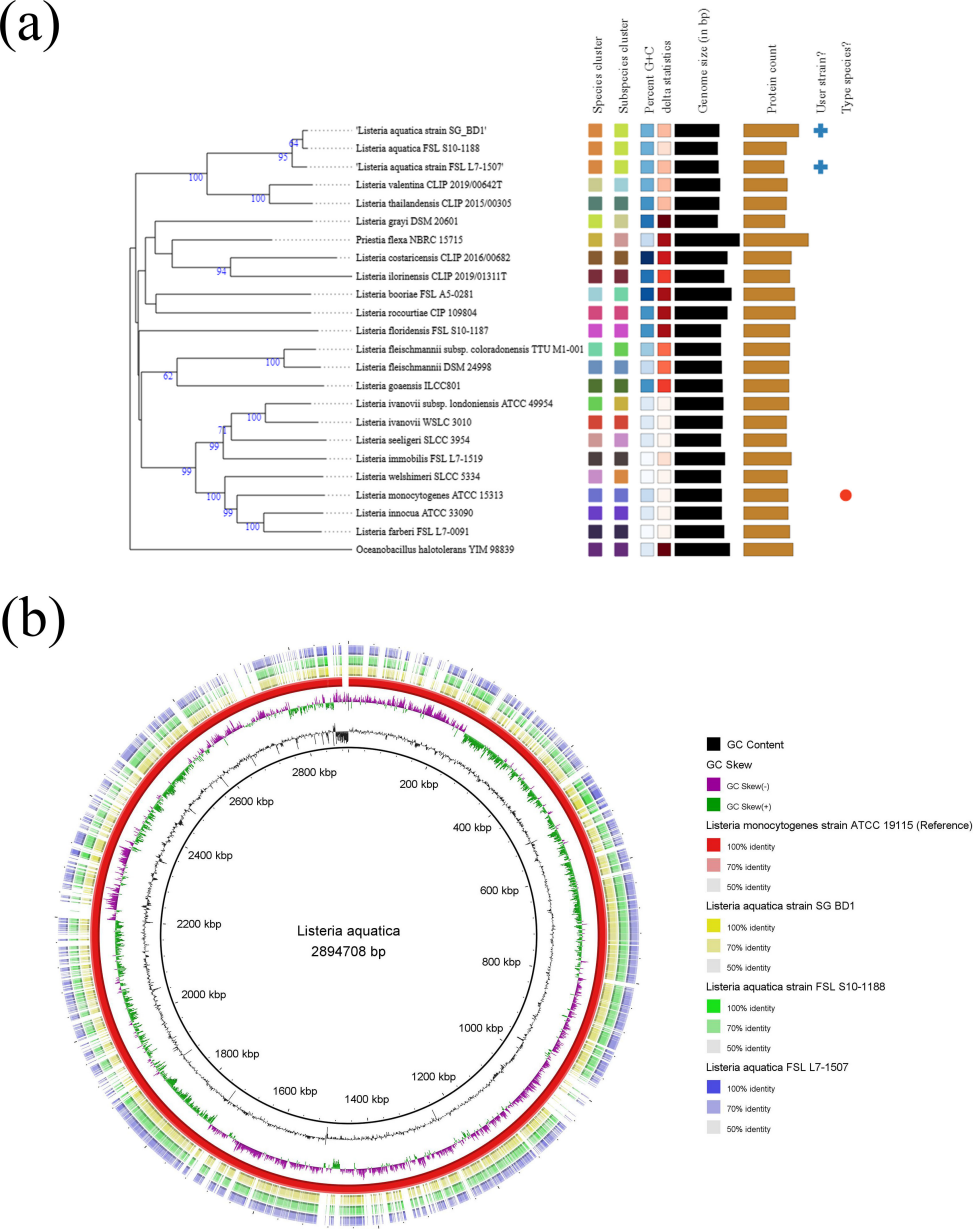
(**a**) Phylogenetic analysis by TYGS shows that the study genome had the highest similarity with *L. aquatica* isolates. The whole genome phylogenetic tree was constructed based on the Genome Blast Distance Phylogeny (GBDP) approach. It inferred with FastME version 2.1.6.1 (15) from GBDP distances calculated from genome sequences. The branch lengths are scaled in terms of GBDP distance formula d5. The numbers above branches are GBDP pseudo-bootstrap support values >60% from 100 replications, with an average branch support of 64.3%. The tree was rooted at the midpoint (16). Here, leaf labels are annotated by affiliation to species clusters, subspecies clusters, percent G + C, delta statistics, genome size (in bp), and protein count (from left to right); (**b**) BRIG tool analysis of the study genome *L. aquatica* strain SG_BD1 (yellow ring) with *L. aquatica* strain FSL S10-1188, *L. aquatica* FSL L7-1507 (green and blue rings, respectively), and the type strain *L. monocytogenes* strain ATCC 19115 (the inner red circle). All sequences were downloaded from the NCBI database.

Assembly yielded a linear genome, 2,690,148 bp in length, consisting of three contigs, a G + C content of 39.6%, and an *N*_50_ value of 2,599,825 bp (Table 1). The sequence coverage of the assembled genome was 99×. Whole genome phylogenetic analysis using the Type Strain Genome Server (TYGS) (13) grouped SG_BD1 with the *L. aquatica* strain isolated from water in Florida, USA (Fig. 1) (1). Analysis using the BLAST Ring Image Generator (BRIG) tool version 0.95 revealed higher similarity of the study genome with two available *L. aquatica* genomes in the NCBI (accession numbers AOCG00000000 and JAARRM000000000), compared to the *L. monocytogenes* strain ATCC 19115 (accession number JARWJJ000000000) (Fig. 1b) (14).

**TABLE 1:** Assembly statistics and important genomic features of the *L. aquatica* strain SG_BD1 from cow dung sample in Bangladesh.

Isolate ID	*L. aquatica* strain SG_BD1
Sample source	Cow dung
Year of isolation	2023
Genome length (bp)	2,690,148
No. of contigs	3
GC content (%)	39.6
*N*_50_ (bp)	2,599,825
Coverage (×)	99
Coding DNA sequence	2,855
tRNA	67
rRNAs (5S, 16S, 23S)	6, 6, 6
Noncoding RNA	4
Antibiotic resistance gene	None
Virulence gene	1 (*clpP*)
CRISPR array	9
Accession No.	JBDILX000000000

PGAP revealed the presence of 2,855 coding DNA sequences, 67 tRNAs, 18 rRNAs, and 4 noncoding RNAs (Table 1). A putative virulence gene *clpP* was demonstrated, which aids in protection from heat stress and intracellular survival in *Listeria* spp. No antibiotic resistance gene was found in the genome, while nine CRISPR arrays were detected (Table 1).

## Data Availability

The whole-genome sequencing effort for *L. aquatica* strain SG_BD1 has been submitted to GenBank with accession number JBDILX00­­0000000 under the BioProject number PRJNA1104809 (BioSample accession number SAMN41088195 and SRA accession number SRR29761655). Additional details can be found here. Accession number of the genomes downloaded from the NCBI: *L. aquatica* FSL S10-1188: AOCG00000000, *L. aquatica* strain FSL L7-1507: JAARRM000000000, and *L. monocytogenes* strain ATCC 19115: JARWJJ000000000.
